# Behavioral betrayal: How select fungal parasites enlist living insects to do their bidding

**DOI:** 10.1371/journal.ppat.1008598

**Published:** 2020-06-18

**Authors:** Brian Lovett, Angie Macias, Jason E. Stajich, John Cooley, Jørgen Eilenberg, Henrik H. de Fine Licht, Matt T. Kasson

**Affiliations:** 1 Division of Plant and Soil Sciences, West Virginia University, Morgantown, West Virginia, United States of America; 2 Department of Microbiology and Plant Pathology and Institute for Integrative Genome Biology, University of California, Riverside, California, United States of America; 3 Department of Ecology and Evolutionary Biology, University of Connecticut, Hartford, Connecticut, United States of America; 4 Department of Plant and Environmental Science, University of Copenhagen, Frederiksberg, Denmark; McGill University, CANADA

“The retention of the power of locomotion is of great importance in so far as the spread of the disease is concerned. It enables the insects not only before the abdomen has ruptured, but even after the fruiting layer has been exposed and, during the time the conidia are being discharged, to wander over the trees and distribute the spores in a far more efficient manner than would be possible if death of the host resulted before the fruiting stage of the fungus was reached—as is usually the case.”—Dr. Alan G. Dustan observing active host transmission by *Entomophthora erupta*-infected *Lygus communis*, 1923 [1].

## What is active host transmission?

Insects under the explicit control of parasitic fungi (entomopathogens) are sometimes characterized by colorful terms, even colloquially categorized as “zombies” [2,3], a moniker that draws comparison to both fictitious and factual elements of contemporary life. Though the effects of entomopathogenic fungi on their hosts are a far cry from behavior-modifying viruses such as rabies or the phantasmic world of brain-eating zombies that drag their way through our popular culture, both rabies and select entomopathogenic fungi are nevertheless archetypal examples of pathogens that actively enlist their living hosts for successful transmission, a phenomenon referred to hereafter as active host transmission (AHT) [4].

Victims of the rabies virus experience hydrophobia, refuse to swallow (allowing the virus to collect around their mouths), and are much more likely to aggressively bite and interact with others [5]. This unsettling rewiring of animal behavior supplants the interests of the victim in favor of the interests of the virus within. The phenomenon of parasite-induced AHT in animal hosts has evolved numerous times across a variety of taxonomic groups. For example, *Toxoplasma gondii*, a protist parasite, suppresses the fear response of rodents and drives them to seek out feline foes to help complete the lifecycle of their protist partner [6]. Horsehair worms (Nematomorpha) encourage their host crickets to drown themselves, which allows these parasites to complete their own lifecycle in water [7]. Likewise, certain entomopathogenic fungi such as *Massospora* spp. manipulate their hosts’ sexual behaviors to increase their odds of transmission [8]. Such engagements appear to serve the interests of the fungal pathogen over the interests of their hosts.

**Fig 1:**
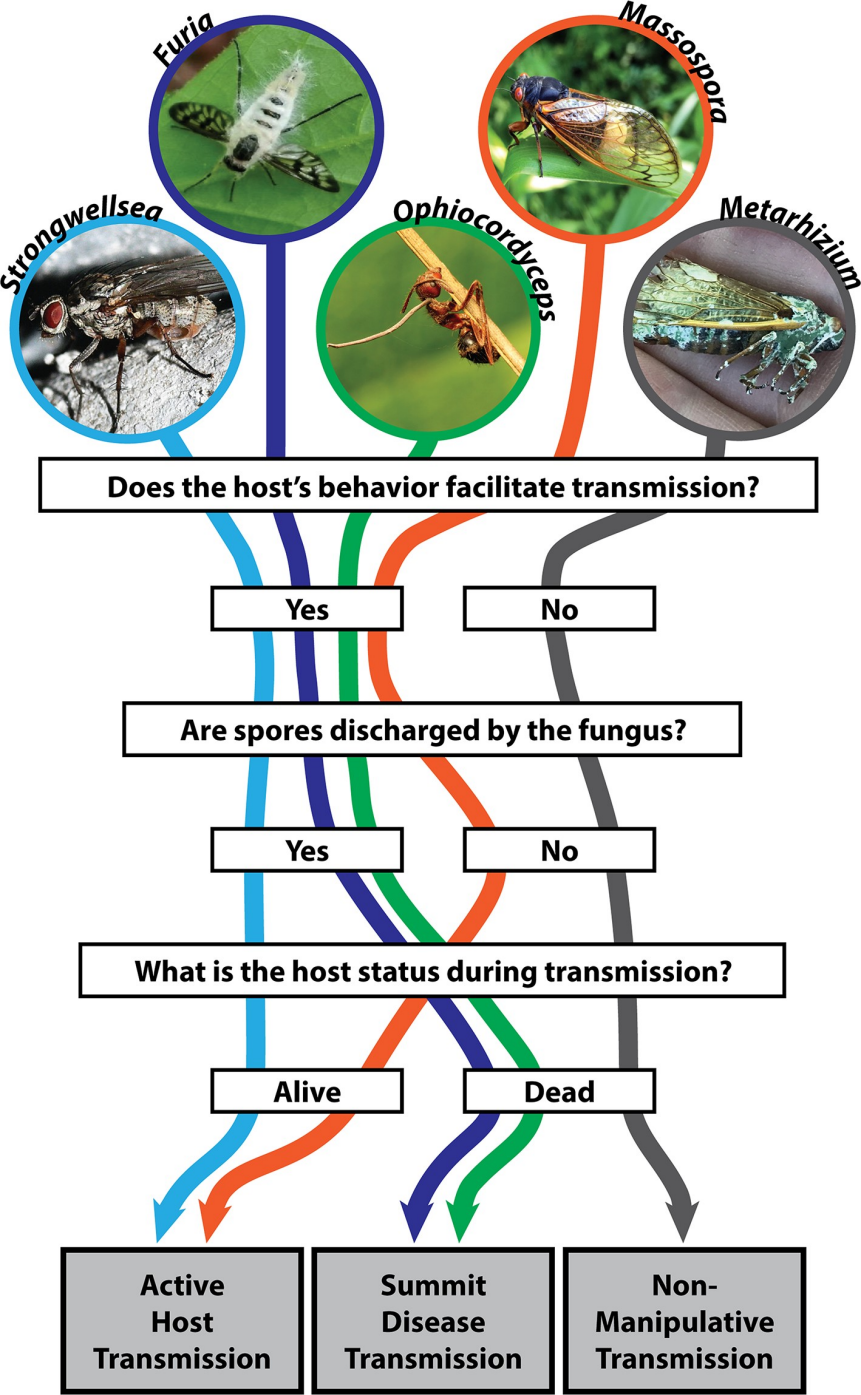
Flowchart of representative entomopathogenic fungi categorized by propensity to manipulate host behavior. This chart illustrates the host behavior, mode of sporulation, and host vitality that delineate which pathogens employ AHT, summit disease, or no behavioral manipulation during infection. The following fungus–host combinations are as follows: *Strongwellsea* aff. *castrans*–*Hylemya vagans*; *Furia ithacensis*–*Rhagionidae*; *Ophiocordyceps* spp.; *Massospora cicadina*–*Magicicada septendecim*; and *Metarhizium anisopliae*–*Magicicada septendecim*. Photo credits to Matt Kasson, except for *Strongwellsea* (credit: Jürgen Peters), *Ophiocordyceps* (credit: Katja Schulz), and *Metarhizium* (credit: John Boback). AHT, active host transmission.

**Fig 2:**
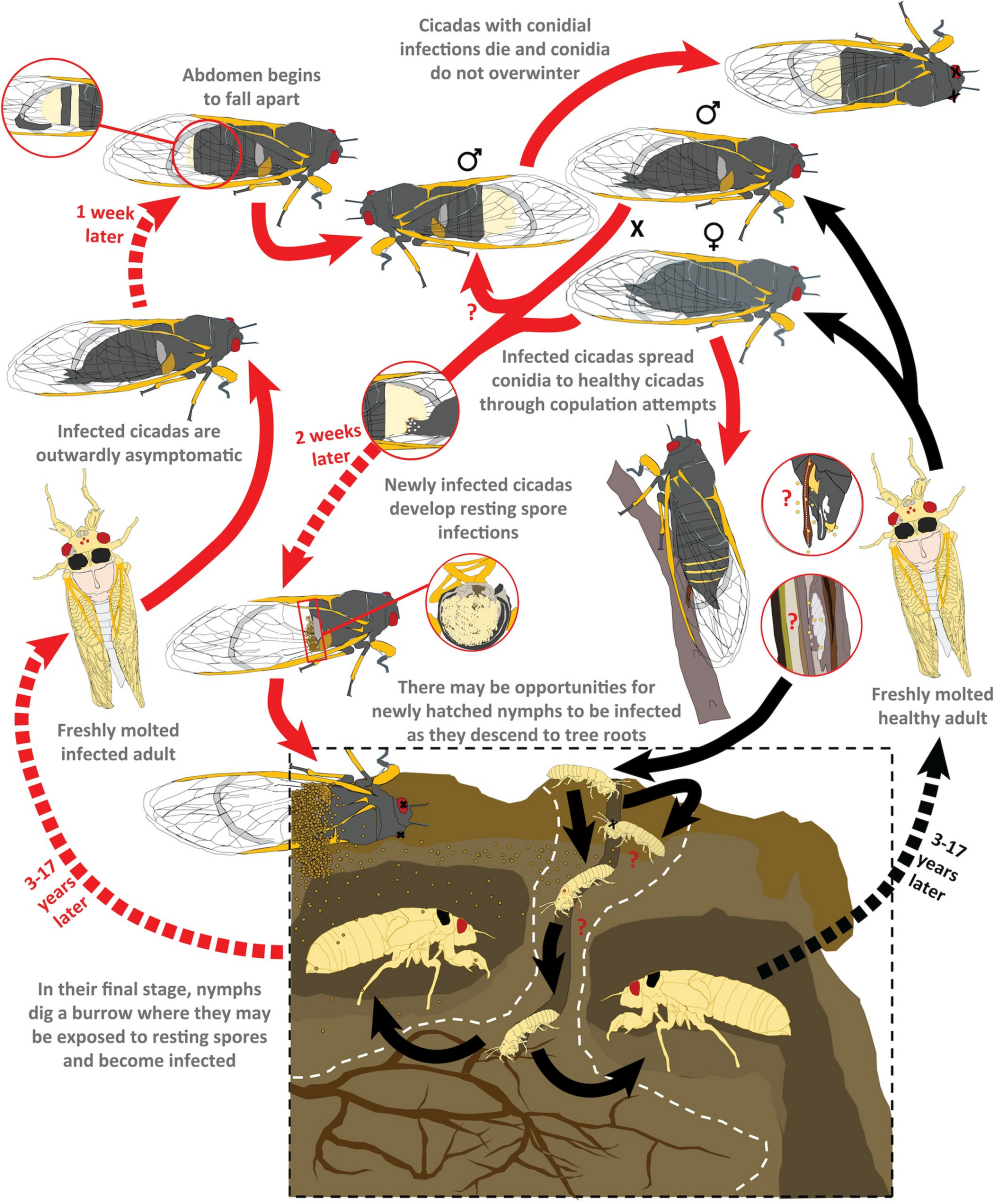
Schematic of *Massospora*–cicada infection cycle. This schematic shows the known and possible modes of infection for cicadas infected with *Massospora* spp. adapted from previous studies on the natural history of this fungal genus [8,13,24,25]. In this figure, the conidial infection is shown in a male for simplicity, but both males and females exhibit conidial infections. Red arrows indicate stages at which cicadas are infected with the fungus, and uncertain routes of infection, with potential significant impacts on the inoculation and spread, are labeled with “?”. Estimates for incubation periods (dashed arrows) for both conidia (typically beginning late May) and resting spore stages (typically first observed in mid-June) are based on the examination of 198 *M*. *cicadina*-infected periodical cicada specimens and 1,236 iNaturalist observations (accessed 16 March 2020) spanning 1974–2019 across 29 states in the mid-Atlantic US. Data for *Massospora* specimens spanned from Texas to Connecticut, with the earliest specimen collected from Mississippi in early May, about a month before they are first found in the northeast.

Manipulation of a host to focus on pathogen transmission is fascinating because it raises questions about the nature of autonomy and shines a light on the physical and behavioral manifestations of parasitism. AHT is a form of biological puppetry in which the pathogen manipulates the behavior of its powerless host. But, identifying clear behavioral manipulations and distinguishing AHT from other notable entomopathogen-induced behaviors such as summit disease, particularly when the infected insects are moribund or dead at the time of their discovery, is a challenge. In anamorphic fungi, including *Metarhizium* species [9], spores are dispersed on contact or passively through the environment. In summit diseases such as *Entomopthora muscae* [3] or *Ophiocordyceps* species [2], dissemination of spores is facilitated by the positioning of the host cadaver. In both of these modes of transmission, spores develop on the mummified host after death, and the deceased host does not actively disperse spores. In contrast, AHT requires 1) a living host and 2) host behavior that facilitates pathogen transmission, thereby increasing pathogen fitness at the expense of host fitness (Fig 1). To achieve these ends, AHT pathogens must produce transmissible reproductive structures while still allowing the host some level of functionality, which is a major distinction between AHT and most other entomopathogenic fungi, in which infectious spores (conidia) are not produced until after host death. Inconspicuous infectious stages also present a challenge for the pathogen itself: developing complex reproductive structures while still inside the living host could result in physical disruption from insect organs, muscles, and exoskeleton that would be static on an insect cadaver. Even when infections are conspicuous, such as when the abdomens of *Massospora*-infected cicadas swell and are eventually shed (Fig 2), the remaining internal organs must retain some functionality to keep the cicada alive. AHT parasites also modify host behaviors so that parasite reproductive structures appear when hosts are manipulated to increase their interactions with uninfected potential hosts. This synchronization could either exploit natural host behaviors or induce behaviors that increase the frequency of interaction between host insects.

## Why haven’t AHT fungi been studied much before?

Recognizing fungi that employ AHT requires comprehensive understanding of the natural history of both the fungal pathogen and its insect host, which is a challenge. Many fungi from the subphylum Entomophthoromycotina are so highly specialized for life on their preferred insect hosts that they can be cultivated beyond a vegetative stage outside of their host only with difficulty (or even not at all), making laboratory studies on transmission, host behavior, etc. based on fungi produced in vitro difficult. Additionally, certain hosts such as periodical cicadas, which have either a 13- or 17-year lifecycle, cannot be practically reared in a lab setting. The infrequent and ephemeral occurrence of other AHT hosts in nature limits our ability to find adequate specimens for formal investigation. Because of these challenges, few actual examples of entomopathogens relying on AHT have been documented. It is likely that others exist but remain undescribed simply because of undersampling. *Massospora* (infecting cicadas) and *Strongwellsea* (infecting flies) species represent the bulk of AHT reports and observations, but a few other examples are known only from the papers in which these fungi are first named, such as *Entomophthora kansana* on calyptrate flies [10], *E*. *erupta* on the black grass bug [1] and the green apple bug [11], and *E*. *thripidum* on thrips [12]. Modern molecular approaches may reveal additional cryptic parasite species that may have been unrecognized because of gaps in our knowledge of host biology or parasite taxonomy [13].

## Is AHT an effective means of dispersal?

Though distinct examples of AHT are few in number, these fungi do not appear to be rare or endangered. This is particularly true for the *Massospora*–cicada system (Fig 2), in which infected individuals can be reliably found in informal surveys of large populations of cicadas [13]. Colonized individuals can be identified in this system when they display a conspicuous infection (i.e., when the abdomen breaks open, revealing a mass of spores); at any given moment, the true number of infected individuals could be much larger because many more individuals may be harboring inconspicuous infections in various stages of progression. A similar challenge is presented by in the *Strongwellsea*–fly system: the fungal pathogen produces a conspicuous hole on the ventral abdomen through which spores are discharged like intrepid parachuters during flight [14]. Until this fungus-lined cavity is observable, it is difficult to distinguish a healthy fly from a *Strongwellsea*-infected victim because this behavioral and bodily modification is more subtle than that observed in *Massospora*-infected cicadas. Still, it is clear that these pathogens are maintained in natural fly populations (from different families) in which they can be identified year after year, notably in Scandinavia [15]. The existence of cyclic AHT infections in diverse insect species suggests that this lifestyle is an effective means of transmission for these host-specific fungal pathogens regardless of whether or not these host–parasite systems are easy to observe.

## How is sexual behavior related to AHT?

Mating involves intimate contact and can thus reliably provide an opportunity for transmitting pathogens; hence, the existence of sexually transmitted infections. The *Massospora*–*Magicicada* parasite–host system functions, in part, as a sexually transmitted infection. Following emergence, healthy periodical cicadas spend several days as nonreceptive adults; after this period, males chorus and females become sexually receptive [16,17]. *Magicicada* sexual behavior is highly stereotyped: males call and females respond with wing flicks, but healthy males never signal with wing flicks [16]. When females remain unmated much beyond the onset of sexual receptivity, their responses become exaggerated with louder, more consistent wing flicks and sometimes even whole-body motions that appear to draw the attention of chorusing males (S1 Text). This exaggerated behavior is a form of hypersexuality that is consistent with age-related decreases in mate choosiness reported in cockroaches [18], medflies [19], parasitoid wasps [20], and other insects (reviewed in [21]). In this context, hypersexuality observed in *Massospora*-infected cicadas is not surprising because infected cicadas remain unmated for their entire lives and may therefore exhibit increased sexual receptivity without any special manipulation by the fungus.

The mechanism by which *Massospora* induces female-associated behaviors in infected male cicadas is unknown [22]. Males with conidia-producing infections (which are spread by close contact among abundant individuals) exhibit sexual behaviors directed at both sexes by additionally wing flicking in response to calls by other males. Since male periodical cicadas with late-season infections (which produce spores that are dispersed into soil) do not wing flick, male wing flicking must therefore result from active manipulation by the fungus during a time when this manipulation directly increases transmission and thus fungal fitness [8]. To our knowledge, this is the only example of AHT in which the pathogen behaves at least in part as a sexually transmitted disease, although natural history studies are lacking.

## What’s driving the evolution of AHT, and did it evolve more than once?

The lifestyles of AHT fungi are clearly contrived, involving both species-specific host disfiguration and incremental production of at least 2 spore types. An outstanding question is how these pathogens have arrived at their current lifestyles; answering this question is particularly difficult because examples of AHT “missing links” have not yet been identified. This could be due to the undersampling that plagues this field, or perhaps the extreme specialization involved makes these taxa vulnerable to extinction. Behavioral manipulation in entomopathogenic fungi is always associated with a high degree of host specificity, and in some sense, AHT represents an endpoint of host specialization. It is noteworthy that entomophthoralean fungi are notorious for having massive genomes full of repetitive DNA, raising the possibility that it is difficult to switch to a generalist lifestyle from their highly specialized lifestyles [23]. Following this line of reasoning, these pathogens may have arrived at their present lifestyle via bursts of genomic innovation that facilitated a high degree of specialization during a brief period of inefficient transmission. This could perhaps have been facilitated by temporarily dense host populations, as occurs in cicadas and certain dipteran species [10].

A recent study found that *Massospora*-infected cicadas contain psychoactive compounds, including cathinone (*Massospora cicadina* in periodical cicadas) and psilocybin (*M*. *levispora*/*platypediae* in annual cicadas) [24]. This study further investigated the phylogenetic relationships of fungi that modify host behavior (both summit disease and AHT entomophthoralean fungi) to conclude that AHT either evolved once or twice and was subsequently lost in multiple or a single summit-disease–causing fungus, respectively, although the phylogenetic placement of several AHT taxa remains unresolved [24]. A single origin for AHT is a tantalizing possibility, but it must be reconciled with the intimate specialization of AHT fungi to quite different hosts [25]. Certainly, AHT fungi could employ similar mechanisms (through bioactive compounds or otherwise) to encourage their disfigured victims to engage in behaviors that increase their contact with other individuals. However, it has not yet been demonstrated that cicadas and flies respond similarly, or at all, to these chemical compounds, and shared physiological cues in holometabolous flies and hemimetabolous cicadas that can be manipulated by their respective AHT fungal pathogens have not yet been identified. Furthermore, not all host insects may have lifestyles in which there are behaviors amenable to manipulation by parasitic fungi.

It is clear that much remains to be discovered about the evolutionary history of AHT in fungi. These questions will be answered with increased sampling, improved genome assemblies, and increased natural history studies into these fascinating fungi and their unlucky insect victims. Despite the challenges of studying AHT fungi, this work will continue apace, spurred by public interest and scientific curiosity.

## Supporting information

S1 Text Personal communication. This personal communication with co-author John Cooley substantiates the aberrant age-related hypersexual behaviors seen in unmated *Magicicada* females.
(DOCX)
