# Supplementary material for: Behavioral betrayal: How select fungal parasites enlist living insects to do their bidding
Source: PLoS Pathog. 2020 Jun 18;16(6):e1008598. doi: 10.1371/journal.ppat.1008598 (PMC7302443; doi:10.1371/journal.ppat.1008598)
Supplement: S1 Text — This personal communication with co-author John Cooley substantiates the aberrant age-related hypersexual behaviors seen in unmated Magicicada females. (DOCX) [file ppat.1008598.s001.docx]

27 April 2020

To Whom It May Concern:

The statement below, from the Lovett *et al*. manuscript “Behavioral Betrayal: how select fungal parasites enlist living insects to do their bidding”:

"When females remain unmated much beyond the onset of sexual receptivity, their responses become exaggerated with louder, more consistent wing flicks and sometimes even whole-body motions that appear to draw the attention of chorusing males (S1 Text). "

Is based on work for my own PhD. thesis (Cooley 1999) and is related to work published in (Cooley and Marshall 2001, Cooley 2004, Cooley et al. 2018). In particular, a research field note digest created 7 September 1998 notes additional behaviors that are examples of age-related hypersexuality:

1. Males typically use vibrating movements of their forelegs to touch females while courting. Females have not normally been observed making these movements; however, if females are prevented from mating, when they age to several days past the typical time they would have mated, they make foreleg vibrating movements on other cicadas that they encounter.
2. Males typically sexually mount females by climbing up on their backs. Females do not normally mount other cicadas; however, if females are prevented from mating, when they age to several days past the typical time they would have mated, they will mount other cicadas and, instead of extending their genitalia, they will extend their ovipositors and attempt to oviposit into the cicada that they are mounting.

Sincerely,

John Cooley

Department of Ecology and Evolutionary Biology

The University of Connecticut

10 South Prospect Street, Hartford CT 06103

959-200-3908

Cooley, J. R. 1999. Sexual behavior in North American cicadas of the genera *Magicicada* and *Okanagana*. Ph.D. The University of Michigan, Ann Arbor.

Cooley, J. R. 2004. Asymmetry and mating success in a periodical cicada, *Magicicada septendecim* (Hemiptera: Cicadidae). Ethology **110**:745-759.

Cooley, J. R., and D. C. Marshall. 2001. Sexual signaling in periodical cicadas, *Magicicada* spp. (Hemiptera: Cicadidae). Behaviour **138**:827-855.

Cooley, J. R., D. C. Marshall, and K. B. R. Hill. 2018. A specialized fungal parasite (Massospora cicadina) hijacks the sexual signals of periodical cicadas (Hemiptera: Cicadidae: Magicicada). Scientific Reports **8**:1432.
